# Risk of hypoglycaemia with insulin degludec versus insulin glargine U300 in insulin-treated patients with type 2 diabetes: the randomised, head-to-head CONCLUDE trial

**DOI:** 10.1007/s00125-019-05080-9

**Published:** 2020-01-27

**Authors:** Athena Philis-Tsimikas, David C. Klonoff, Kamlesh Khunti, Harpreet S. Bajaj, Lawrence A. Leiter, Melissa V. Hansen, Lone N. Troelsen, Steen Ladelund, Simon Heller, Thomas R. Pieber, on behalf of the CONCLUDE Study Group

**Affiliations:** 1 Scripps Whittier Diabetes Institute, 10140 Campus Point Drive, Suite 200, San Diego, CA 92121 USA; 2 Diabetes Research Institute, Mills-Peninsula Medical Center, San Mateo, CA USA; 3 Diabetes Research Centre, University of Leicester, Leicester, UK; 4 LMC Diabetes and Endocrinology, Brampton, ON Canada; 5 Li Ka Shing Knowledge Institute, Division of Endocrinology & Metabolism, St Michael’s Hospital, University of Toronto, Toronto, ON Canada; 6 Novo Nordisk A/S, Søborg, Denmark; 7 Academic Unit of Diabetes, Endocrinology and Metabolism, University of Sheffield, Sheffield, UK; 8 Division of Endocrinology and Diabetology, Department of Internal Medicine, Medical University of Graz, Graz, Austria

**Keywords:** Clinical science, Hypoglycaemia, Insulin degludec, Insulin glargine, Insulin therapy

## Abstract

**Aims/hypothesis:**

A head-to-head randomised trial was conducted to evaluate hypoglycaemia safety with insulin degludec 200 U/ml (degludec U200) and insulin glargine 300 U/ml (glargine U300) in individuals with type 2 diabetes treated with basal insulin.

**Methods:**

This randomised (1:1), open-label, treat-to-target, multinational trial included individuals with type 2 diabetes, aged ≥18 years with HbA_1c_ ≤80 mmol/mol (9.5%) and BMI ≤45 kg/m^2^. Participants were previously treated with basal insulin with or without oral glucose-lowering drugs (excluding insulin secretagogues) and had to fulfil at least one predefined criterion for hypoglycaemia risk. Both degludec U200 and glargine U300 were similarly titrated to a fasting blood glucose target of 4.0–5.0 mmol/l. Endpoints were assessed during a 36 week maintenance period and a total treatment period up to 88 weeks. There were three hypoglycaemia endpoints: (1) overall symptomatic hypoglycaemia (either severe, an event requiring third-party assistance, or confirmed by blood glucose [<3.1 mmol/l] with symptoms); (2) nocturnal symptomatic hypoglycaemia (severe or confirmed by blood glucose with symptoms, between 00:01 and 05:59 h); and (3) severe hypoglycaemia. The primary endpoint was the number of overall symptomatic hypoglycaemic events in the maintenance period. Secondary hypoglycaemia endpoints included the number of nocturnal symptomatic events and number of severe hypoglycaemic events during the maintenance period.

**Results:**

Of the 1609 randomised participants, 733 of 805 (91.1%) in the degludec U200 arm and 734 of 804 (91.3%) in the glargine U300 arm completed the trial (87.3% and 87.8% completed on treatment, respectively). Baseline characteristics were comparable between the two treatment arms. For the primary endpoint, the rate of overall symptomatic hypoglycaemia was not significantly lower with degludec U200 vs glargine U300 (rate ratio [RR] 0.88 [95% CI 0.73, 1.06]). As there was no significant difference between treatments for the primary endpoint, the confirmatory testing procedure for superiority was stopped. The pre-specified confirmatory secondary hypoglycaemia endpoints were analysed using pre-specified statistical models but were now considered exploratory. These endpoints showed a lower rate of nocturnal symptomatic hypoglycaemia (RR 0.63 [95% CI 0.48, 0.84]) and severe hypoglycaemia (RR 0.20 [95% CI 0.07, 0.57]) with degludec U200 vs glargine U300.

**Conclusions/interpretation:**

There was no significant difference in the rate of overall symptomatic hypoglycaemia with degludec U200 vs glargine U300 in the maintenance period. The rates of nocturnal symptomatic and severe hypoglycaemia were nominally significantly lower with degludec U200 during the maintenance period compared with glargine U300.

**Trial registration:**

ClinicalTrials.gov NCT03078478

**Funding:**

This trial was funded by Novo Nordisk (Bagsvaerd, Denmark)

**Electronic supplementary material:**

The online version of this article (10.1007/s00125-019-05080-9) contains peer-reviewed but unedited supplementary material, which is available to authorised users.

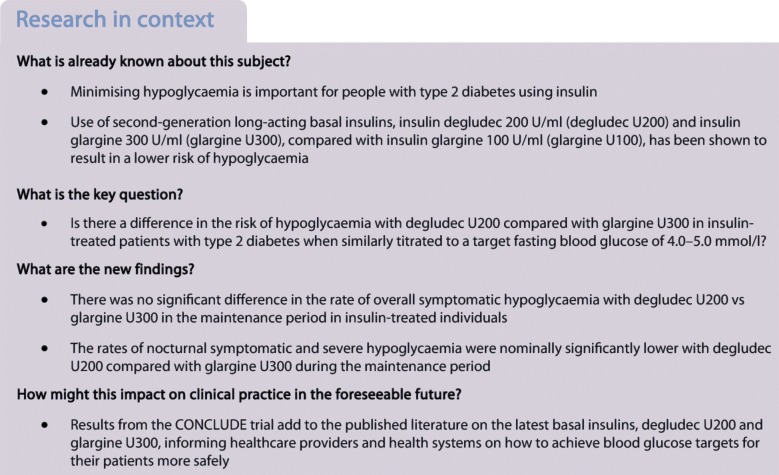



## Introduction

Hypoglycaemia is a known complication of insulin treatment and is acknowledged as the main limiting factor for achieving tight glycaemic control [1, 2]. The two most recently developed second-generation, longer-acting basal insulins, insulin degludec and insulin glargine 300 U/ml (glargine U300), have flatter and more stable steady-state pharmacokinetic and pharmacodynamic profiles compared with long-acting insulin glargine 100 U/ml (glargine U100) [3–6]. Insulin degludec has a lower day-to-day variability in glucose-lowering effect compared with glargine U100 and glargine U300 [6, 7], whereas there are contradictory reports regarding within-day variability when comparing insulin degludec and glargine U300 [7, 8].

Insulin degludec and glargine U300 have been shown to be associated with a lower risk of hypoglycaemia, at equivalent glycaemic control compared with glargine U100 in individuals with type 2 diabetes [9–20]. Glargine U300 is a concentrated formulation of glargine U100 and has also been shown to be as effective as glargine U100 in terms of glycaemic control in individuals with type 2 diabetes, but with a higher (12–14%) basal insulin dose requirement [14–20]. Recent results in insulin-naive individuals with type 2 diabetes revealed similar HbA_1c_ reductions for insulin degludec and glargine U300 [21]. This trial also reported a similar overall risk of hypoglycaemia between the two insulins and a lower rate of hypoglycaemia in the titration period with glargine U300 vs insulin degludec, while no evaluation of severe hypoglycaemia was conducted as only one event was recorded during the trial. In addition, the dose of insulin degludec was lower than the dose of glargine U300 at the end of the trial by 0.11 U/kg.

The primary objective of the Trial Comparing the Efficacy and Safety of Insulin Degludec and Insulin Glargine 300 Units/ml in Subjects with Type 2 Diabetes Mellitus Inadequately Treated with Basal Insulin and Oral Antidiabetic Drugs (CONCLUDE), a randomised head-to-head clinical trial, was to investigate the effect of insulin degludec 200 U/ml (degludec U200) and glargine U300 on hypoglycaemia in insulin-treated individuals with type 2 diabetes.

## Methods

### Trial design

Detailed methods of CONCLUDE have been described previously [22]. Briefly, this was a treat-to-target, randomised, open-label, active comparator-controlled trial that was conducted at 229 sites in 11 countries. The original 58 week trial duration comprised 52 weeks of active treatment with designation of the first 16 weeks as the titration period and the remaining 36 weeks as the maintenance period (hereafter referred to as the ‘variable maintenance period’). In February 2018, a protocol amendment led to the extension of the trial, resulting in a total trial duration of up to 94 weeks with up to 88 weeks of active treatment, including a new maintenance period (hereafter referred to as the ‘maintenance period’) of 36 weeks. A detailed rationale for this amendment has been published previously [22] and the key reasons for the amendment are outlined in ESM Fig. 1. In brief, routine monitoring of blinded data showed an unusual pattern in the reporting of glycaemic variables and hypoglycaemic events. Specifically, the glycaemic data were inconsistent between central-laboratory-measured variables (HbA_1c_ and fasting plasma glucose [FPG]) and patient-reported fasting self-measured blood glucose (SMBG) values. Data available from SMBG monitoring indicated to the patient that the blood glucose levels were higher than they actually were, potentially increasing the risk of hypoglycaemia as a result of unnecessary insulin up-titration. At the time of the amendment, the number of patient-reported hypoglycaemic events confirmed by blood glucose was low while the number of pseudo-hypoglycaemic events (blood glucose >3.9 mmol/l with symptoms) was high compared with the SWITCH 2 trial (comparing the effect of insulin degludec vs insulin glargine U100 on in individuals with type 2 diabetes) [10]. These observations, seen in general across the entire trial population, were related to the glycaemic data collection system (MyGlucoHealth blood glucose meter and electronic diary). Therefore, because of these safety concerns, the glycaemic data collection system was discontinued during the variable maintenance period. This system was replaced with an Abbott blood glucose meter and paper diary to be used for the remainder of the trial. To accommodate these changes, preserve the scientific integrity of the trial and ensure sufficient data collection for the confirmatory endpoints using the same glycaemic data collection system (Abbott blood glucose meter and paper diary), a new 36 week maintenance period was included in the trial. At the time of the amendment, recruitment had been finalised and all participants on treatment had completed the titration period. The duration of the variable maintenance period was dependent on each participant’s individual randomisation date and/or approval of the amended protocol by health authorities and local ethics committees, if applicable. After implementation of the amended protocol, participants were asked to come in and initiate the maintenance period as soon as the resources were available at the trial site, irrespective of the next planned visit. Thus, all participants were not required to have all visits scheduled between weeks 16 and 52. The trial data remained blinded at the point of discovering the issue with the glycaemic data collection system and the implementation of the protocol amendment. No unplanned interim analysis of the trial data from the titration period was conducted. The primary endpoint (number of severe or blood-glucose-confirmed symptomatic hypoglycaemic events) at the completion of the maintenance period was evaluated utilising the same analysis duration and statistical methods as the original protocol. Changes were implemented to maintain participant safety and protect the scientific integrity of the trial.

CONCLUDE is registered with ClinicalTrials.gov no. NCT03078478. The trial was conducted in accordance with the Declaration of Helsinki and ICH Good Clinical Practice Guideline [23, 24]. The protocol was approved by independent ethics committees or institutional review boards for each centre; written informed consent was obtained from each participant before any trial-related activities.

### Participants and treatments

Eligible participants included adults aged >18 years with type 2 diabetes with HbA_1c_ ≤80 mmol/mol (9.5%), BMI ≤45 kg/m^2^ and treated with basal insulin (once or twice daily; NPH insulin, insulin detemir, glargine U100) with or without oral glucose-lowering drugs (OADs) at stable doses (any combination of metformin, dipeptidyl peptidase-4 inhibitor, α-glucosidase inhibitor, thiazolidinedione and sodium–glucose cotransporter 2 inhibitor) for at least 90 days. In addition, participants had to fulfil at least one risk criterion for hypoglycaemia [22]. The main exclusion criteria were treatment with bolus or premixed insulin or with sulfonylureas/glinides within 90 days before the screening visit, severe renal impairment (eGFR <30 ml min^−1^ [1.73 m]^−2^), or impaired liver function (alanine aminotransferase or aspartate aminotransferase ≥2.5 times the upper limit of normal).

Consenting participants were randomised using a trial-specific, interactive-voice, web-response system. Participants were randomised 1:1 to receive degludec U200 (Novo Nordisk, Bagsvaerd, Denmark; the 100 U/ml and 200 U/ml concentrations of degludec are bioequivalent and interchangeable [25–27]) or glargine U300 (Sanofi, Paris, France) administered once daily. Within each treatment arm, participants were randomised 1:1 to administer basal insulin either in the morning (from waking to breakfast) or in the evening (from main evening meal to bedtime). The same dosing time was maintained for each participant throughout the trial. When initiating degludec U200, the pre-trial daily basal insulin dose was reduced by 20%, as per the protocol, irrespective of prior insulin type. Glargine U300 was initiated according to its label: unit-to-unit switch for participants on once-daily basal insulin; 20% reduction for those on twice-daily NPH insulin (US patients) or any twice-daily basal insulin (European and Canadian patients). The insulin dose was titrated similarly for both insulins: once-weekly titration was based on the mean of three pre-breakfast SMBG measurements, with a fasting blood glucose target of 4.0–5.0 mmol/l. The insulin dose was adjusted in multiples of 2 U ranging from −4 U to +8 U depending on the mean pre-breakfast SMBG level [22]. The type and dose of pre-trial OADs remained unchanged throughout the trial unless safety reasons required a change.

### Endpoints

The primary endpoint was the rate of overall symptomatic hypoglycaemic events (defined as severe [an event requiring third-party assistance [28]] or confirmed blood glucose <3.1 mmol/l [with symptoms]) during the maintenance period. Secondary confirmatory hypoglycaemia endpoints included the rate of nocturnal symptomatic hypoglycaemic events (severe or blood-glucose-confirmed with symptoms, occurring between 00:01 and 05:59 h) and the rate of severe hypoglycaemic events during the maintenance period. Overall symptomatic, nocturnal symptomatic and severe hypoglycaemic events were also assessed during the total treatment period (up to 88 weeks) as secondary endpoints. Other secondary endpoints included change from baseline to end of treatment in HbA_1c_ level and FPG level, basal insulin dose at the end of treatment, pre-breakfast SMBG level and body weight. The composite endpoints HbA_1c_ <53 mmol/mol (7.0%) with no overall symptomatic hypoglycaemia and HbA_1c_ <53 mmol/mol (7.0%) with no nocturnal symptomatic hypoglycaemia were assessed during the maintenance period. The number of adverse events between the two treatment arms was also assessed during the trial period. An independent external event adjudication committee validated the following selected adverse events in a blinded manner: fatal events and severe hypoglycaemia.

### Statistical analysis

The statistical analyses of the primary and secondary endpoints have been described previously [22]. Endpoints related to hypoglycaemia and safety endpoints were summarised using the safety analysis set; efficacy endpoints were summarised using the full analysis set. Statistical superiority testing of the primary and confirmatory secondary endpoints was performed following a hierarchical testing procedure to control the family-wise type I error rate in the strong sense and has been described previously [22]. The sample size was calculated to ensure at least 80% power for the primary endpoint analysis.

A negative binomial model with pre-trial OADs, region, sex and dosing time as fixed effects, age as covariate and logarithm of the exposure time as offset was used to estimate the rate ratio (RR) of hypoglycaemic events during the maintenance and total treatment periods. Participants with no on-treatment data during the maintenance period had values imputed for the maintenance period analyses based on participants discontinuing treatment during the maintenance period. Multiple imputations were performed using standard methods aligned with the analyses and planned to create 1000 complete datasets. The results were then combined using Rubin’s methods [29]. The proportion of participants experiencing hypoglycaemic events was analysed post hoc using a logistic regression model. The model included treatment, pre-trial OADs, region, sex and dosing time as fixed effects and age as a covariate, and logarithm of the exposure time as offset. Change from baseline to end of treatment in HbA_1c_ levels, FPG levels, SMBG and body weight were analysed post hoc using mixed models for repeated measures (MMRM) with treatment, pre-trial OADs, region, sex and dosing time as fixed effects, and age and baseline HbA_1c_/FPG as covariates. Pre-specified sensitivity analyses were also conducted to test the primary and protocol-specified confirmatory secondary hypoglycaemia endpoints without imputed data as well as capping the number of hypoglycaemic events at three. Further post hoc sensitivity analyses controlling for variation across sites were conducted for HbA_1c_ and FPG.

## Results

### Participants

**Fig. 1:**
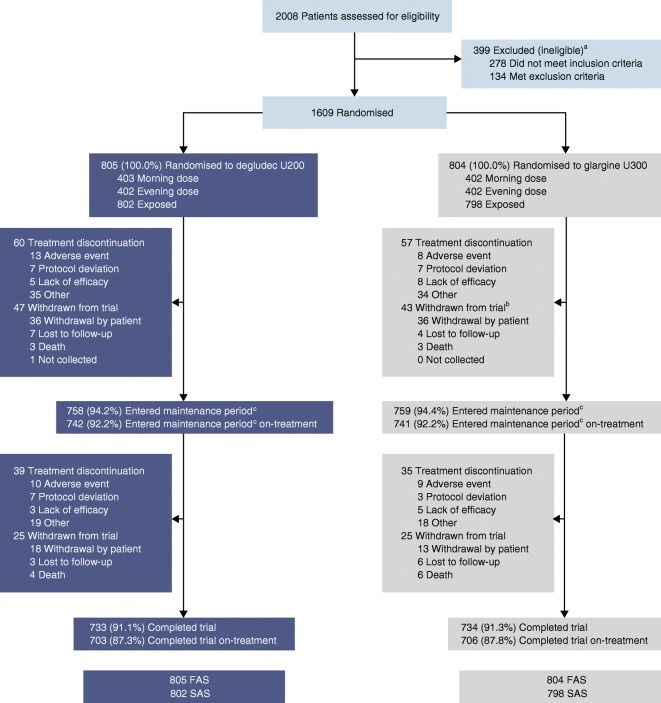
Patient disposition. ^a^Some participants fulfilled more than one inclusion or exclusion criterion. ^b^Two additional participants discontinued treatment before the protocol amendment and neither withdrew nor re-consented. ^c^New, 36 week maintenance period (52–88 weeks). The number of participants who entered the maintenance period = (participants randomised) – (participants withdrawn from the trial). The number of participants who entered the maintenance period on-treatment = (participants exposed) – (participants who discontinued treatment). The number of participants who completed trial = (participants who entered the maintenance period) – (participants withdrawn from the trial). The number of participants who completed trial on-treatment = (participants who entered the maintenance period on treatment) – (participants who discontinued treatment). Exposed was defined as ‘randomised and received treatment’. The number of participants that discontinued treatment includes the number that withdrew from the trial. FAS, full analysis set; SAS, safety analysis set

Of the 2008 eligible participants screened, 1609 were randomised to receive either degludec U200 (*n* = 805) or glargine U300 (*n* = 804) (Fig. 1). A total of 1467 participants (91.2%) completed the trial of whom 1409 (87.6%) completed the trial on treatment. The proportion of participants withdrawing from the trial and discontinuing treatment prematurely was similar for both treatment groups. The protocol amendment did not have an apparent impact on participant retention rates (Fig. 1).

The characteristics of the participants at baseline were similar in the two treatment groups (Table 1) and did not differ between the randomised population and those entering the maintenance period (ESM Table 1). The mean age was 62.8 years, the mean duration of diabetes was 15.1 years, and the mean±SD HbA_1c_ level was 59.2 ± 10.5 mmol/mol (7.6 ± 1.0%). At screening, most participants were using glargine U100 (65.0%) and were treated with metformin (77.5%).

**Table 1:** Baseline characteristics of participants

Characteristic	Degludec U200 (*N* = 805)	Glargine U300 (*N* = 804)	*p* value
Age, years	62.9 ± 10.0	62.8 ± 10.0	0.8599
Men	472 (58.6)	436 (54.2)	0.0785
Hispanic or Latino ethnicity	84 (10.4)	100 (12.4)	0.2111
Race			0.6978
White	693 (86.1)	699 (86.9)	
Black or African-American	78 (9.7)	65 (8.1)	
Asian	25 (3.1)	29 (3.6)	
Other	9 (1.1)	11 (1.4)	
Diabetes duration, years	15.1 ± 8.2	15.0 ± 8.4	0.7676
Oral glucose-lowering treatment^a^	715 (88.8)	708 (88.1)	0.5835
Metformin	622 (77.3)	625 (77.7)	
Dipeptidyl peptidase-4 inhibitor	178 (22.1)	152 (18.9)	
SGLT-2 inhibitor	150 (18.6)	153 (19.0)	
Combination of glucose-lowering treatments^b^	41 (5.1)	44 (5.5)	
Thiazolidinedione	37 (4.6)	25 (3.1)	
α-Glucosidase inhibitors	6 (0.7)	2 (0.2)	
Basal insulin	803 (99.8)^c^	804 (100.0)	0.1014
Detemir	171 (21.2)	139 (17.3)	
Glargine U100	505 (62.7)	541 (67.3)	
NPH insulin	127 (15.8)	124 (15.4)	
Basal insulin dose, U	42.7 ± 29.5	42.2 ± 29.1	0.7077
Body weight, kg	91.6 ± 18.1	90.6 ± 17.9	0.2396
BMI, kg/m^2^	31.7 ± 5.3	31.5 ± 5.2	0.5119
HbA_1c_, mmol/mol	59.0 ± 10.8	59.4 ± 10.2	0.5137
HbA_1c_, %	7.6 ± 1.0	7.6 ± 0.9	0.5137
FPG, mmol/l	7.9 ± 2.6	8.0 ± 2.6	0.6205
eGFR based on CKD-EPI^d^, ml min^−1^ [1.73 m]^−2^	78.8 ± 21.2	80.0 ± 20.6	0.2422
Participants fulfilling ≥1 of the following hypoglycaemia risk inclusion criteria			
≥1 severe hypoglycaemic event within the last year	50 (6.2)	48 (6.0)	
Moderate chronic renal failure	152 (18.9)	132 (16.4)	
Hypoglycaemia symptom unawareness	166 (20.6)	141 (17.5)	
Exposed to insulin for ≥5 years	406 (50.4)	391 (48.6)	
Hypoglycaemic event within last 12 weeks	466 (57.9)	479 (59.6)	

Data are for the full analysis set and are shown as *n* (%) or mean±SD; percentage refers to the proportion of participants on degludec U200 or glargine U300 treatment. The *p* value was determined by two-sided test of no difference

^a^One participant on sulfonylurea was randomised in error and discontinued treatment

^b^The combinations of glucose-lowering treatments includes allowed combinations, as per the inclusion criteria, only

^c^One participant who was on premix NPH insulin and one patient who was insulin-naive were randomised in error

^d^Taken at screening

CKD-EPI, Chronic Kidney Disease Epidemiology Collaboration; SGLT-2, sodium–glucose cotransporter 2

### Hypoglycaemia endpoints

#### Overall symptomatic hypoglycaemia

**Fig. 2:**
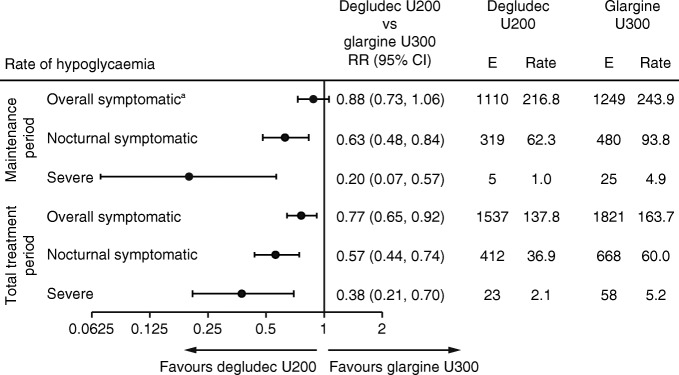
The rate of hypoglycaemia. Overall symptomatic hypoglycaemia was defined as severe hypoglycaemia (an event requiring third-party assistance as per the ADA definition [28]) or blood glucose <3.1 mmol/l confirmed with symptoms. Nocturnal symptomatic hypoglycaemia was defined as severe hypoglycaemia or blood glucose <3.1 mmol/l confirmed with symptoms, occurring between 00:01 and 05:59 h. ^a^Primary endpoint. E, events; rate, events per 100 person-years of observation

For the primary endpoint, overall symptomatic hypoglycaemia, the rate was not significantly lower with degludec U200 compared with glargine U300 during the maintenance period (RR 0.88 [95% CI 0.73, 1.06]) (Fig. 2). Because there was no significant difference between treatments for the primary endpoint, the confirmatory testing procedure for superiority was stopped. The pre-specified confirmatory secondary hypoglycaemia endpoints, nocturnal symptomatic and severe hypoglycaemia during the maintenance period, could not be controlled for the family-wise type I error and therefore were now considered exploratory. The sensitivity analyses conducted to test the primary endpoint without imputed data and capping the number of hypoglycaemic events at three showed similar results to the main analysis (ESM Table 2).

**Fig. 3:**
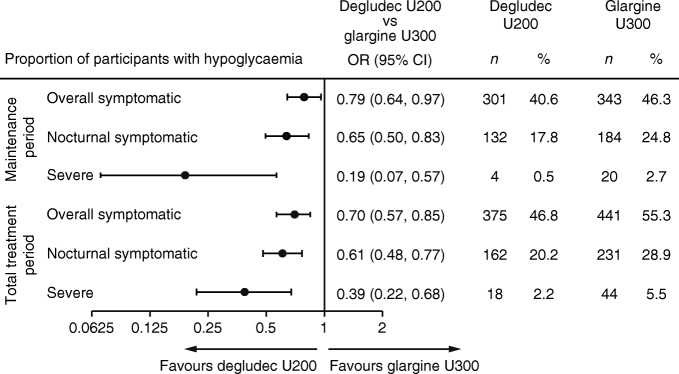
The proportion of participants with hypoglycaemia (post hoc). Overall symptomatic hypoglycaemia was defined as severe hypoglycaemia (an event requiring third-party assistance as per the ADA definition [28]) or blood glucose <3.1 mmol/l confirmed with symptoms. Nocturnal symptomatic hypoglycaemia was defined as severe hypoglycaemia or blood glucose <3.1 mmol/l confirmed with symptoms, occurring between 00:01 and 05:59 h. %, proportion of participants with events; *n*, number of participants experiencing events

The proportion of participants experiencing overall symptomatic hypoglycaemia during the maintenance period was lower for those treated with degludec U200 (40.6%) compared with glargine U300 (46.3%): OR 0.79 (95% CI 0.64, 0.97), post hoc analysis (Fig. 3). During the total treatment period, the rate and the proportion of participants (post hoc) experiencing overall symptomatic hypoglycaemia was lower with degludec U200 vs glargine U300 (Figs 2 and 3).

#### Nocturnal symptomatic hypoglycaemia

The rate of nocturnal symptomatic hypoglycaemia was lower with degludec U200 compared with glargine U300 during the maintenance period (RR 0.63 [95% CI 0.48, 0.84]) (Fig. 2). The sensitivity analyses conducted to test this endpoint without imputed data and capping the number of hypoglycaemic events at three showed similar results (ESM Table 2). The proportion of participants during the maintenance period experiencing nocturnal symptomatic hypoglycaemia was lower for those treated with degludec U200 (17.8%) compared with glargine U300 (24.8%): OR 0.65 (95% CI 0.50, 0.83), post hoc analysis (Fig. 3). Similar results were observed during the total treatment period for the rate and the proportion of participants (post hoc) experiencing nocturnal symptomatic hypoglycaemia (Figs 2 and 3).

#### Severe hypoglycaemia

The rate of severe hypoglycaemia was lower with degludec U200 compared with glargine U300 during the maintenance period (RR 0.20 [95% CI 0.07, 0.57]) (Fig. 2). The sensitivity analyses conducted to test this endpoint without imputed data and capping the number of hypoglycaemic events at three showed similar results (ESM Table 2). In addition, the proportion of participants experiencing severe hypoglycaemia was lower for those treated with degludec U200 (0.5%) than for those treated with glargine U300 (2.7%): OR 0.19 (95% CI 0.07, 0.57), post hoc analysis (Fig. 3). Similar results were observed during the total treatment period for the rate and the proportion of participants (post hoc) experiencing severe hypoglycaemia (Figs 2 and 3).

#### Hypoglycaemia during titration and variable maintenance periods

**Fig. 4:**
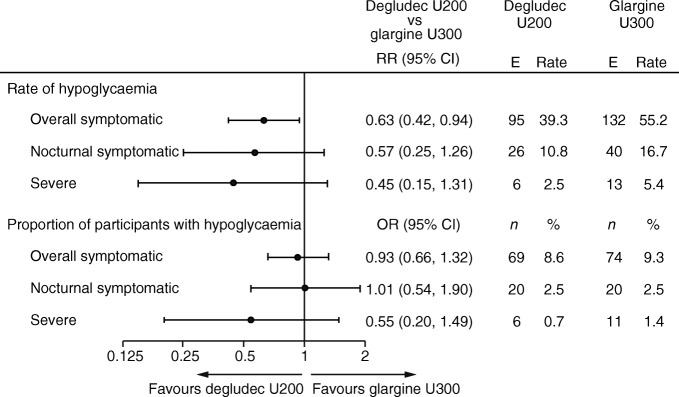
Hypoglycaemia endpoints during the titration period. Overall symptomatic hypoglycaemia was defined as severe hypoglycaemia (an event requiring third-party assistance as per the ADA definition [28]) or blood glucose <3.1 mmol/l confirmed with symptoms. Nocturnal symptomatic hypoglycaemia was defined as severe hypoglycaemia or blood glucose <3.1 mmol/l confirmed with symptoms, occurring between 00:01 and 05:59 h. %, proportion of participants with events; E, events; *n*, number of participants with events; rate, events per 100 person-years of observation

**Fig. 5:**
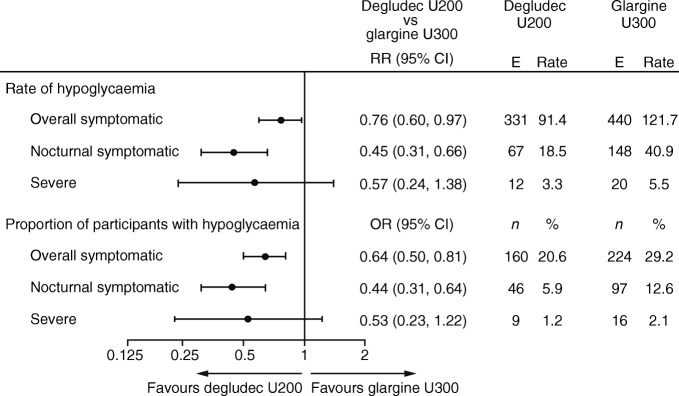
Hypoglycaemia endpoints during the variable maintenance period. Overall symptomatic hypoglycaemia was defined as severe hypoglycaemia (an event requiring third-party assistance as per the ADA definition [28]) or blood glucose <3.1 mmol/l confirmed with symptoms. Nocturnal symptomatic hypoglycaemia was defined as severe hypoglycaemia or blood glucose <3.1 mmol/l confirmed with symptoms, occurring between 00:01 and 05:59 h. %, proportion of participants with events; E, events; *n*, number of participants with events; rate, events per 100 person-years of observation

The rates and the proportions of participants (post hoc) experiencing hypoglycaemia during the titration and variable maintenance periods are shown in Figs 4 and 5.

### Glycaemic control

**Fig. 6:**
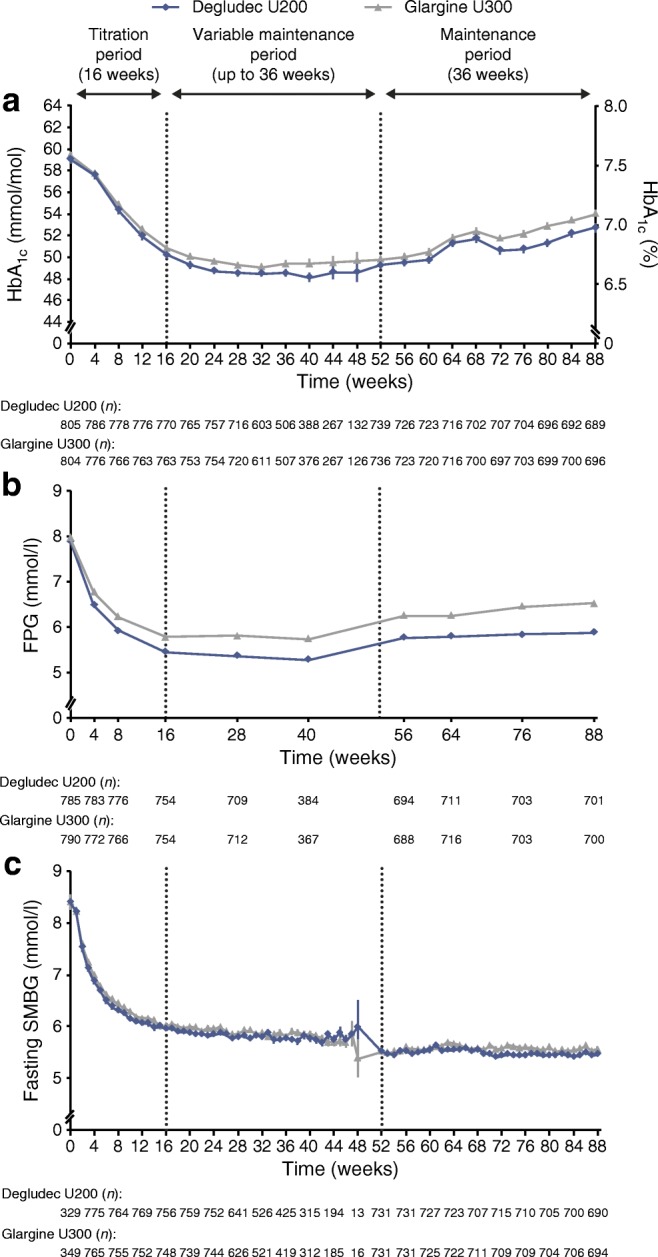
HbA_1c_, FPG and fasting SMBG over time. (**a**) HbA_1c_ over the study period. Estimated treatment difference from baseline to end of treatment for degludec U200 vs glargine U300 was −1.07 mmol/mol (95% CI −1.94, −0.20) (−0.10% [95% CI −0.18, −0.02]). (**b**) FPG over the study period. Estimated treatment difference from baseline to end of treatment for degludec U200 vs glargine U300 was −0.62 mmol/l (95% CI −0.82, −0.43). (**c**) SMBG over the study period. Estimated treatment difference from baseline to end of treatment for degludec U200 vs glargine U300 was −0.18 mmol/l (95% CI −0.37, 0.01). Data are presented as mean±SEM, with the number of participants (*n*) shown below each graph. Vertical dotted lines illustrate the end of the titration period (week 16) and the beginning of the maintenance period (week 52). According to the protocol, all participants were not required to complete all visits in the variable maintenance period and therefore the number of participants at each week decreased during this period

The observed mean HbA_1c_ values at the end of the titration period were 50.2 mmol/mol (6.8%) with degludec U200 and 50.9 mmol/mol (6.8%) with glargine U300. At the end of treatment, the observed mean HbA_1c_ was 52.8 mmol/mol (7.0%) and 54.1 mmol/mol (7.1%) for degludec U200 and glargine U300, respectively. An analysis of HbA_1c_ demonstrated a reduction in the HbA_1c_ level from baseline to the end of treatment with degludec U200 compared with glargine U300: observed mean −5.90 mmol/mol (−0.54%) vs −5.04 mmol/mol (−0.46%); estimated treatment difference −1.07 mmol/mol (95% CI −1.94, −0.20) (−0.10% [95% CI −0.18, −0.02]), post hoc analysis (Fig. 6a). At the end of treatment, the observed mean FPG was 5.9 mmol/l and 6.5 mmol/l for degludec U200 and glargine U300, respectively. There was also a reduction in FPG from baseline to the end of treatment with degludec U200 compared with glargine U300: observed mean −1.97 mmol/l vs −1.43 mmol/l; estimated treatment difference −0.62 mmol/l (95% CI −0.82, −0.43), post hoc analysis (Fig. 6b). Sensitivity analyses controlling for variation across study sites were conducted for HbA_1c_ and FPG and showed similar results to the main analysis (ESM Table 3). Over 88 weeks, pre-breakfast SMBG values were similar in the two treatment groups, decreasing during the titration period and then levelling off (estimated treatment difference −0.18 mmol/l [95% CI −0.37, 0.01], post hoc analysis) (Fig. 6c).

At the end of the maintenance period, 35.3% of participants treated with degludec U200 vs 30.0% of participants treated with glargine U300 achieved a composite endpoint of HbA_1c_ <53 mmol/mol (7.0%) with no overall symptomatic hypoglycaemia (OR 1.31 [95% CI 1.04, 1.65], post hoc analysis). Similarly, 47.4% of participants treated with degludec U200 achieved an HbA_1c_ <53 mmol/mol (7.0%) with no nocturnal symptomatic hypoglycaemia compared with 39.3% of participants treated with glargine U300 (OR 1.23 [95% CI 0.99, 1.54], post hoc analysis).

### Insulin dose

**Fig. 7:**
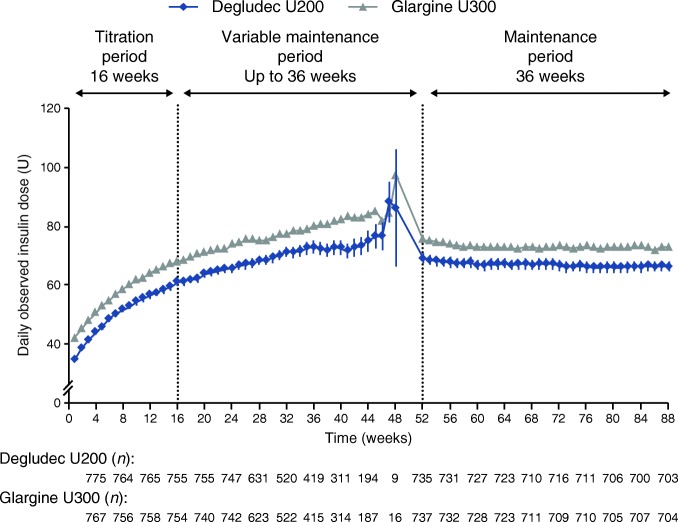
Basal insulin dose over time. Data are presented as mean±SEM, with the number of participants (*n*) shown below the graph. Vertical dotted lines illustrate the end of the titration period (week 16) and the beginning of the maintenance period (week 52). According to the protocol, all participants were not required to complete all visits in the variable maintenance period and therefore the number of participants at each week decreased during this period

The observed mean±SD baseline insulin dose for the degludec U200 and glargine U300 treatment arms was 42.7 ± 29.5 U and 42.2 ± 29.1 U, respectively. At the start of treatment, the observed mean±SD basal insulin dose was 35.1 ± 23.8 U in the degludec U200 group and 42.4 ± 29.2 U in the glargine U300 group. At the end of treatment, the observed mean±SD dose was 66.6 ± 48.5 U for the degludec U200 group and 73.0 ± 48.5 U for the glargine U300 group (Fig. 7).

### Adverse events and body weight

The number of adverse events per 100 person-years of exposure (PYE) was 367.3 in the degludec U200 group and 365.4 per 100 PYE in the glargine U300 group; the corresponding rate of serious adverse events was 27.3 per 100 PYE vs 25.7 per 100 PYE, respectively (ESM Table 4). The most frequent adverse events (≥5%) reported were nasopharyngitis, upper respiratory tract infection and diarrhoea. Serious adverse events attributed to hypoglycaemia during the trial were relatively infrequent (8 events [0.72 events/100 PYE] for degludec U200 and 21 events [1.89 events/100 PYE] for glargine U300). A total of seven participants (all on treatment) died in the degludec U200 group and nine (six on treatment) died in the glargine U300 group, of which no deaths were attributed to hypoglycaemic events or the trial products.

At the end of treatment, the observed change from baseline in body weight was higher in the degludec U200 group compared with the glargine U300 group (mean±SD: 2.9 ± 5.2 kg vs 1.7 ± 5.8 kg), with an estimated treatment difference of 1.18 kg (95% CI 0.60, 1.75; post hoc analysis).

## Discussion

In this open-label, randomised, treat-to-target trial in individuals with type 2 diabetes treated with insulin, administration of degludec U200 resulted in no significant difference in the rate of overall symptomatic hypoglycaemia but it did result in a nominally significantly lower rate of nocturnal symptomatic and severe hypoglycaemia during the maintenance period compared with glargine U300. In the total treatment period, the rate of hypoglycaemia was lower with degludec U200 for all three hypoglycaemia endpoints compared with glargine U300. Similarly, post hoc analyses showed that a lower proportion of participants experienced all three hypoglycaemia endpoints during the maintenance and total treatment periods. These hypoglycaemia results were achieved in the present trial without compromising glycaemic control.

Although the rate of overall symptomatic hypoglycaemia during the maintenance period (primary endpoint) was lower with degludec U200 compared with glargine U300, the difference did not reach statistical significance. Thus, it was not possible to rule out the possibility of no effect of degludec U200 compared with glargine U300. However, the 95% CI for the RR, from 0.73 to 1.06, indicates no clinically significant harm with degludec U200.

The BRIGHT trial was the first randomised clinical trial that compared degludec U100 with glargine U300 in insulin-naive individuals [21]. The results from BRIGHT demonstrated a comparable primary outcome of glycaemic control along with similar rates and proportions of participants experiencing hypoglycaemia (any-time and nocturnal) with glargine U300 vs degludec U100 in both the total treatment period (24 weeks) and the maintenance period (12 weeks). However, a lower rate and proportion of participants experienced any-time hypoglycaemia during the titration period (12 weeks) with glargine U300 than with degludec U100. The same results for the titration period were not observed in CONCLUDE. Because BRIGHT was undertaken in insulin-naive individuals without a history of severe hypoglycaemia or hypoglycaemia unawareness, there were not enough severe hypoglycaemic events to evaluate this outcome. The higher rates of severe hypoglycaemic events in the insulin-experienced population of CONCLUDE, who had at least one hypoglycaemia risk factor (including a history of severe hypoglycaemia), permitted an evaluation of these events. In terms of the trial design, CONCLUDE had longer durations of trial, titration period and maintenance period than BRIGHT. The two trials also had different primary endpoints: hypoglycaemia for CONCLUDE and HbA_1c_ for BRIGHT. Furthermore, the two trials used different definitions of hypoglycaemia with different blood glucose levels (<3.1 mmol/l for CONCLUDE vs ≤3.9 mmol/l or <3.0 mmol/l for BRIGHT) and had different titration algorithms. Target fasting SMBG in BRIGHT was 4.4–5.6 mmol/l, while in CONCLUDE the target was 4.0–5.0 mmol/l. Finally, individuals treated with sulfonylureas were excluded from CONCLUDE while they were included in BRIGHT (65.7% of participants at baseline).

Previous studies have demonstrated that glargine U300 has a weight benefit compared with glargine U100 [15–20]. During the CONCLUDE trial a greater increase in body weight was observed with degludec U200 compared with glargine U300.

This trial has several limitations. The requirement to amend the protocol and include an additional maintenance period added complexity to the trial. However, the change in protocol and trial design (i.e. the change in the glycaemic data collection system) was essential to protect the safety of the trial participants. Ultimately, we believe that these protocol revisions did not impact the scientific integrity of the trial because few participants (*n* = 25 [1.6%]) did not re-consent for the new maintenance period (between the day that the protocol amendment was implemented and each participant’s individual day of initiating the new maintenance period; the remaining participants were withdrawn or lost to follow-up prior to the protocol amendment). However, we cannot exclude that unknown confounding factors could have been introduced that may have biased the perception of the investigators and participants, possibly affecting the conduct of the trial. In addition, it is important to note that although the MyGlucoHealth meter led to inaccurate blood glucose measurements, all participants used this system for the entire titration period thus the data reporting pattern was the same for both treatment groups. Moreover, the reporting of severe hypoglycaemia was not influenced by the inaccurate measurement, as these events were classified according to the ADA definition (requiring third-party assistance) and were externally adjudicated throughout the trial. Furthermore, degludec U200 and glargine U300 were compared in a controlled clinical trial setting, which limits the generalisability to routine clinical practice where individuals may have chronically higher HbA_1c_ levels despite basal insulin use, receive less support to prevent hypoglycaemia as well as use different titration targets and have issues regarding adherence. However, a recent literature review found that rates of hypoglycaemia overlap substantially in real-world settings and clinical trial settings [30]. Finally, data concerning the sociodemographic aspects (such as occupation, education level, etc.), which could potentially impact hypoglycaemia, were not collected during the trial.

Our trial has several strengths, including the large enrolment of insulin-treated individuals with a long duration of diabetes. The CONCLUDE population represents a more accurate reflection of patients seen in clinical practices than most published insulin randomised controlled trials where individuals with hypoglycaemia risk factors are typically excluded [9, 14–21]. Furthermore, the duration of the trial was relatively long, compared with most other clinical trials in this patient population, with a total treatment period of up to 88 weeks. This allowed for the assessment of glycaemic and hypoglycaemia outcomes over a longer time period than other trials.

In conclusion, the rate of overall symptomatic hypoglycaemia with degludec U200 was not significantly lower than with glargine U300 in the maintenance period. The rate of nocturnal symptomatic and severe hypoglycaemia were nominally significantly lower with degludec during the maintenance period compared with glargine U300.

## Electronic supplementary material

ESM (PDF 233 kb)

## Data Availability

The data generated during and/or analysed during the current trial are available from the corresponding author on reasonable request.

## Abbreviations

Degludec U200: Insulin degludec 200 U/ml
FPG: Fasting plasma glucose
Glargine U100: Insulin glargine 100 U/ml
Glargine U300: Insulin glargine 300 U/ml
MMRM: Mixed model of repeated measures
OAD: Oral glucose-lowering drug
PYE: Person-years of exposure
RR: Rate ratio
SMBG: Self-measured blood glucose
