# Supplementary material for: Risk of hypoglycaemia with insulin degludec versus insulin glargine U300 in insulin-treated patients with type 2 diabetes: the randomised, head-to-head CONCLUDE trial
Source: Diabetologia. 2020 Jan 27;63(4):698–710. doi: 10.1007/s00125-019-05080-9 (PMC7054369; doi:10.1007/s00125-019-05080-9)
Supplement: Supplementary file 1 — (PDF 233 kb) [file 125_2019_5080_MOESM1_ESM.pdf]

Supplementary appendix

Risk of hypoglycaemia with insulin degludec versus insulin glargine U300 in insulin-treated patients with type 2 diabetes: the randomised, head-to-head CONCLUDE trial

Athena Philis-Tsimikas, David C. Klonoff, Kamlesh Khunti, Harpreet S. Bajaj, Lawrence A. Leiter, Melissa Voigt Hansen, Lone Nørgård Troelsen, Steen Ladelund, Simon Heller, Thomas R. Pieber, on behalf of the CONCLUDE Study Group.

Contents

ESM Table 1: Baseline characteristics of participants entering the maintenance period 2

ESM Table 2. Sensitivity analyses of hypoglycaemic endpoints 4

ESM Table 3. Sensitivity analysis of glycaemic endpoints to control for variation across sites 5

ESM Table 4: Treatment-emergent adverse events (total treatment period) 6

ESM Fig. 1: Trial design 7

ESM Table 1: Baseline characteristics of participants entering the maintenance period

	**Degludec U200 (*N*=758)**	**Glargine U300 (*N*=759)**	***p*-value[e]**
Age (years)	62.7 ± 10.0	62.6 ± 10.0	0.7994
Men	443 (58.4)	409 (53.9)	0.0786
Ethnicity			0.2557
Hispanic or Latino	78 (10.3)	93 (12.3)	
Race			0.7417
White	656 (86.5)	662 (87.2)	
Black or African American	70 (9.2)	61 (8.0)	
Asian	23 (3.0)	26 (3.4)	
Other	9 (1.2)	10 (1.3)	
Diabetes duration (years)	15.0 ± 8.1	14.8 ± 8.2	0.6597
Oral antihyperglycaemic treatment[a]	671 (88.5)	668 (88.0)	0.5872
Metformin	587 (77.4)	594 (78.3)	
Dipeptidyl-peptidase-4 inhibitors	169 (22.3)	141 (18.6)	
SGLT-2 inhibitors	144 (19.0)	147 (19.4)	
Combinations of antihyperglycaemic treatments[b]	38 (5.0)	39 (5.1)	
Thiazolidinedione	34 (4.5)	23 (3.0)	
Alpha-glucosidase inhibitors	3 (0.4)	2 (0.3)	
Basal insulin	756 (99.7)[c]	759 (100.0)	0.1080
Detemir	162 (21.4)	131 (17.3)	
Glargine U100	474 (62.5)	508 (66.9)	
NPH insulin	120 (15.8)	120 (15.8)	
Basal insulin dose (U)	42.2 ± 28.5	41.8 ± 29.0	0.7913
Body weight (kg)	91.6 ± 17.9	90.7 ± 17.9	0.3008
BMI (kg/m^2)	31.7 ± 5.2	31.5 ± 5.2	0.5408
HbA_{1c} (%)	7.5 ± 1.0	7.6 ± 0.9	0.5523
HbA_{1c} (mmol/mol)	58.9 ± 10.8	59.2 ± 10.1	0.5523
FPG (mmol/l)	7.9 ± 2.6	7.9 ± 2.6	0.7102
eGFR (ml min^{-1} [1.73 m]$^{-2}$) based on CKD-EPI[d]	79.2 ± 20.9	80.3 ± 20.5	0.2703
Participants with hypoglycaemia risk inclusion criteria			–
Fulfilling ≥1 of the following criteria	546 (72.0)	515 (67.9)	
≥1 severe hypoglycaemic event within the last year	48 (6.3)	47 (6.2)	
Moderate chronic renal failure	135 (17.8)	120 (15.8)	

Hypoglycaemia symptom unawareness	157 (20.7)	132 (17.4)	
Exposed to insulin for ≥5 years	379 (50.0)	364 (48.0)	
Hypoglycaemic event within last 12 weeks	446 (58.8)	456 (60.1)	

Data are for the full analysis set. Data listed are number (proportion [%]) or mean ± standard deviation. Percentage refers to the proportion of participants on degludec U200 or glargine U300 treatment.

[a]One participant on sulfonylurea was randomised in error and discontinued treatment.

[b]The combinations of antihyperglycaemic treatments includes allowed combinations, as per the inclusion criteria, only.

[c]One participant who was on premix NPH insulin and one participant who was insulin-naive were randomised in error.

[d]Taken at screening.

[e]Two-sided test of no difference.

CKD-EPI, Chronic Kidney Disease Epidemiology Collaboration; FPG, fasting plasma glucose; glargine U100, insulin glargine 100 units/ml; glargine U300, insulin glargine 300 units/ml; NPH, neutral protamine Hagedorn; SGLT-2, sodium-glucose co-transporter-2.

ESM Table 2. Sensitivity analyses of hypoglycaemic endpoints

**Without imputed data**	**Rate ratio (95% CI)**
Overall symptomatic hypoglycaemia	0.82 (0.67, 1.00)
Nocturnal symptomatic hypoglycaemia	0.63 (0.47, 0.84)
Severe hypoglycaemia	0.20 (0.07, 0.59)
**Capped at 3 hypoglycaemic events**	**Rate ratio (95% CI)**
Overall symptomatic hypoglycaemia	0.89 (0.77, 1.03)
Nocturnal symptomatic hypoglycaemia	0.63 (0.49, 0.81)
Severe hypoglycaemia	0.21 (0.07, 0.58)

Overall symptomatic: severe or blood glucose (<3.1 mmol/l)-confirmed with symptoms. Nocturnal symptomatic: severe or blood glucose (<3.1 mmol/l)-confirmed with symptoms, occurring between 00:01 and 05:59. Severe: an event requiring third party assistance as per the American Diabetes Association definition.

ESM Table 3. Sensitivity analysis of glycaemic endpoints to control for variation across sites

	Treatment difference (95% CI)
HbA_{1c} (%)	–0.10 (–0.17, –0.02)
HbA_{1c} (mmol/mol)	–1.05 (–1.89, –0.21)
FPG (mmol/l)	–0.62 (–0.80, –0.44)

FPG, fasting plasma glucose.

ESM Table 4: Treatment-emergent adverse events (total treatment period)

	Degludec U200 (*N*=802) PYE=1115.7				Glargine U300 (*N*=798) PYE=1112.7			
	n	**%**	**Events**	**Rate**	***n***	**%**	**Events**	**Rate**
Adverse events	683	85.2	4098	367.3	650	81.5	4066	365.4
Serious adverse events	167	20.8	305	27.3	141	17.7	286	25.7
Severe adverse events	123	15.3	218	19.5	120	15.0	220	19.8
Adverse events probably related to trial product	55	6.9	78	7.0	55	6.9	67	6.0
Fatal events	7	0.9	9	0.8	6	0.8	8	0.7

Data are for the safety analysis set.

%, proportion of participants with events; glargine U300, insulin glargine 300 units/ml; n, number of participants with events; PYE, patient-years of exposure; rate, events per 100 patient-years of exposure.

ESM Fig. 1: Trial design

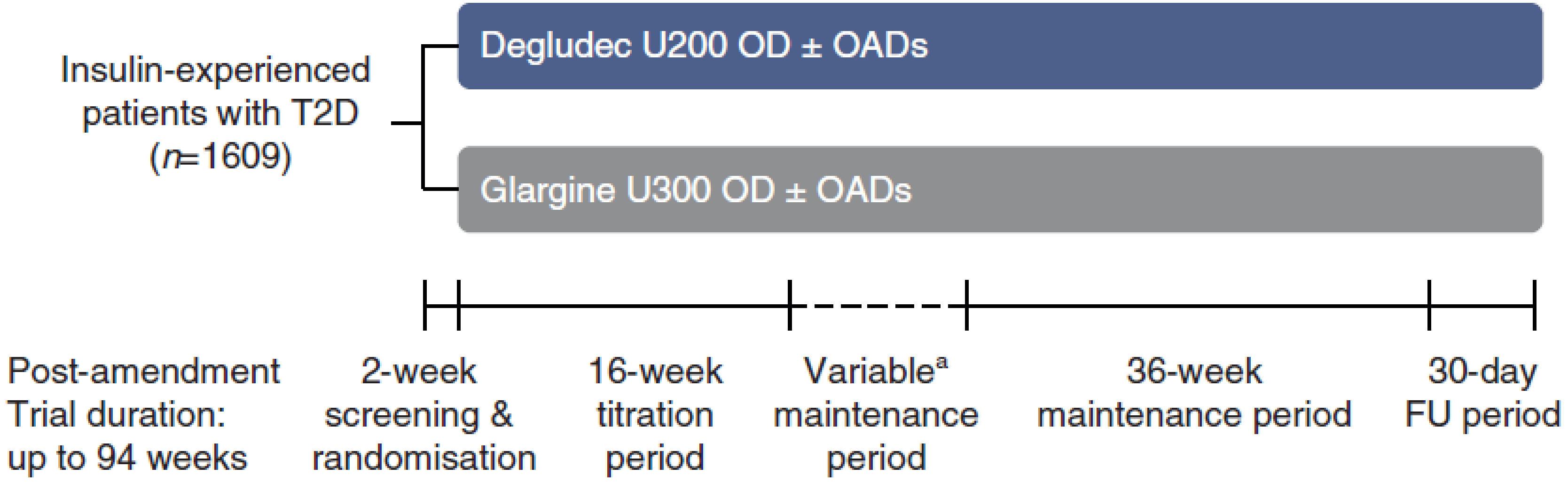

[a]Routine medical monitoring activities during the trial revealed an unusual and potentially unsafe reporting pattern of glycaemic levels and hypoglycaemic events. The data indicated that these patterns were related to the glycaemic data collection system (MyGlucoHealth blood glucose meter and an electronic diary). A major protocol amendment was implemented to discontinue and replace the glycaemic data collection system. At the time of the amendment, recruitment had been finalised and all participants on-treatment had completed the titration period. The duration of the variable maintenance period was dependent on each participant's individual randomisation date and/or approval of the amended protocol by health authorities and local ethics committees, if applicable. After implementation of the amended protocol, participants were asked to come in and initiate the maintenance period as soon as the resources were available at the trial site irrespective of the next planned visit, whereby all participants received a standard Abbott blood glucose meter and paper diary to be used for the remainder of the trial. Thus, all participants were not required to have all visits scheduled between weeks 16 and 52.
FU, follow-up; glargine U300, insulin glargine 300 units/ml; OAD, oral glucose-lowering drug; OD, once daily; T2D, type 2 diabetes.
